# Supplementary material for: Visual statistical learning and integration of perceptual priors are intact in attention deficit hyperactivity disorder
Source: PLoS One. 2020 Dec 17;15(12):e0243100. doi: 10.1371/journal.pone.0243100 (PMC7746270; doi:10.1371/journal.pone.0243100)
Supplement: S1 File — (PDF) [file pone.0243100.s001.pdf]

Visual statistical learning and integration of perceptual priors are intact in Attention Deficit Hyperactivity Disorder

Katie L. Richards, Povilas Karvelis, Stephen M. Lawrie, & Peggy Seriès

Supplementary Material

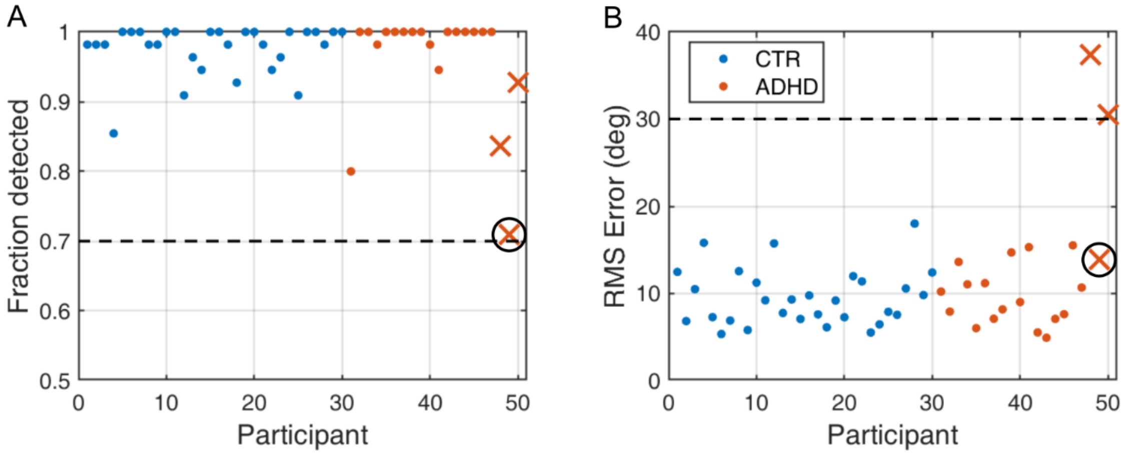

Supplementary Figure 1. Performance on high contrast trials: detection rate (left) and root mean square error (RMSE) (right). Dashed lines indicate the exclusion criteria (70% detection and 30° RMSE). Participants who did not satisfy at least one of these criteria are denoted with a cross marker and were excluded from further analysis. In addition, one more participant was excluded (circled) due to poor detection performance on low-contrast trials (<50%). This resulted in 20 controls (CTR) and 17 attention deficit hyperactivity disorder (ADHD) participants being included in the analysis.

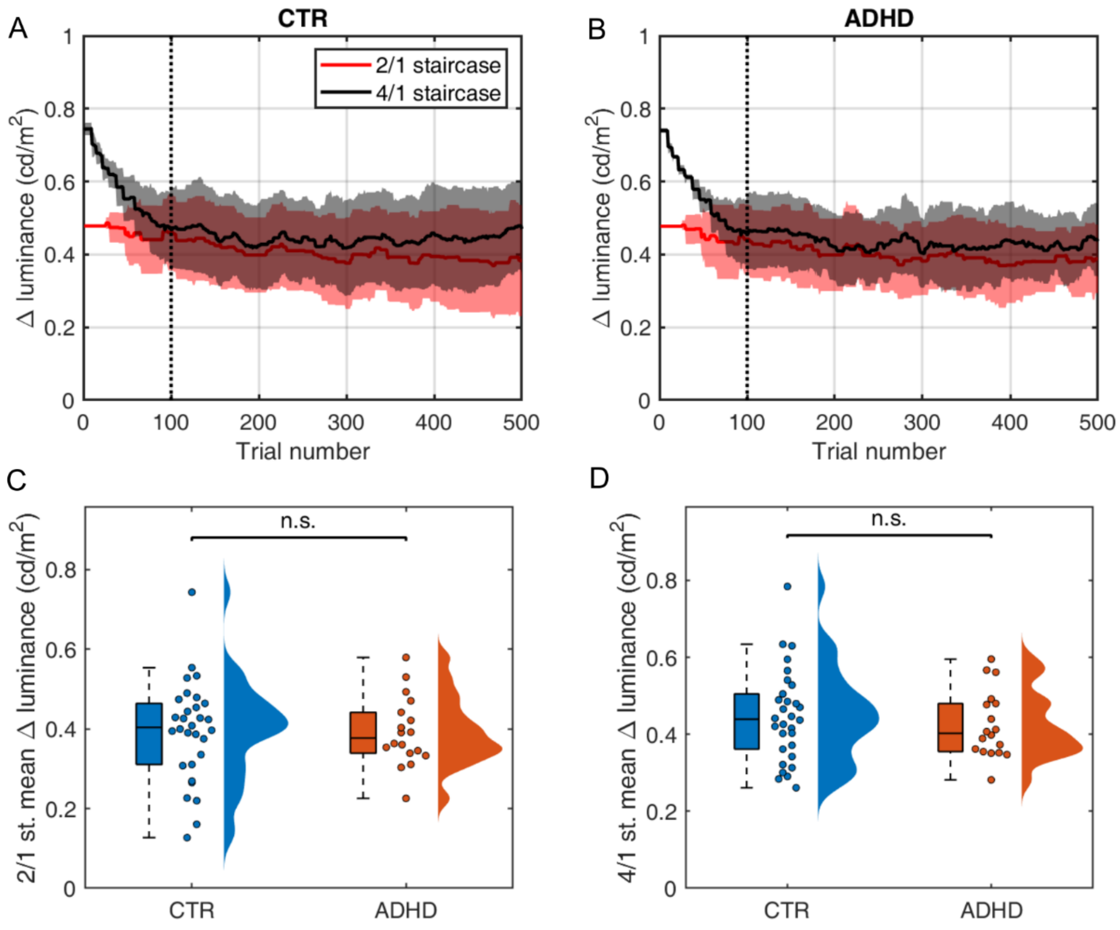

Supplementary Figure 2. 2/1 and 4/1 staircase contrast levels throughout the task. (A) Controls, (B) ADHD. Both groups were found to reach stable contrast levels after 100 trials. These trials were removed from further data analysis. The groups did not differ in the achieved contrast levels (C, D). n.s. = non-significant

Emergence of prior effects

We investigated at which point in the task the acquired expectations started to have a significant effect on performance and whether this was different for participants with ADHD. To do so, we computed cumulative moving averages at every 55 trials for different measures of interest and tested for significance of the prior effects (**Supplementary Fig. 3**). In the order presented, we tested when the estimation reaction times (RT) at ±32° became shorter than at all other directions (Supp. Fig.3A), when the detection at ±32° became higher than at all other directions (Supp. Fig.3B), when the bias at

±64° become more negative then bias at ±32° (Supp. Fig.3C), and when the probability of hallucinating within 16° of ±32° became larger than at other directions (p_ratio; Eq. (6)) (Supp. Fig.3D). For all measures we performed one-tailed Wilcoxon signed rank test, pooling data across the groups to test for the effects of the prior, and two-tailed Wilcoxon rank-sum test for comparing the groups at each step of 55 trials.

We found that the effects of the acquired priors became significant within 110 trials for all measures, while group differences were largely not significant, except for detection performance, where ADHD group showed stronger effects of the acquired priors towards the end of the task, and for estimation bias, where the ADHD group showed less estimation bias between trials 220 to 330 (**Supplementary Fig. 3**).

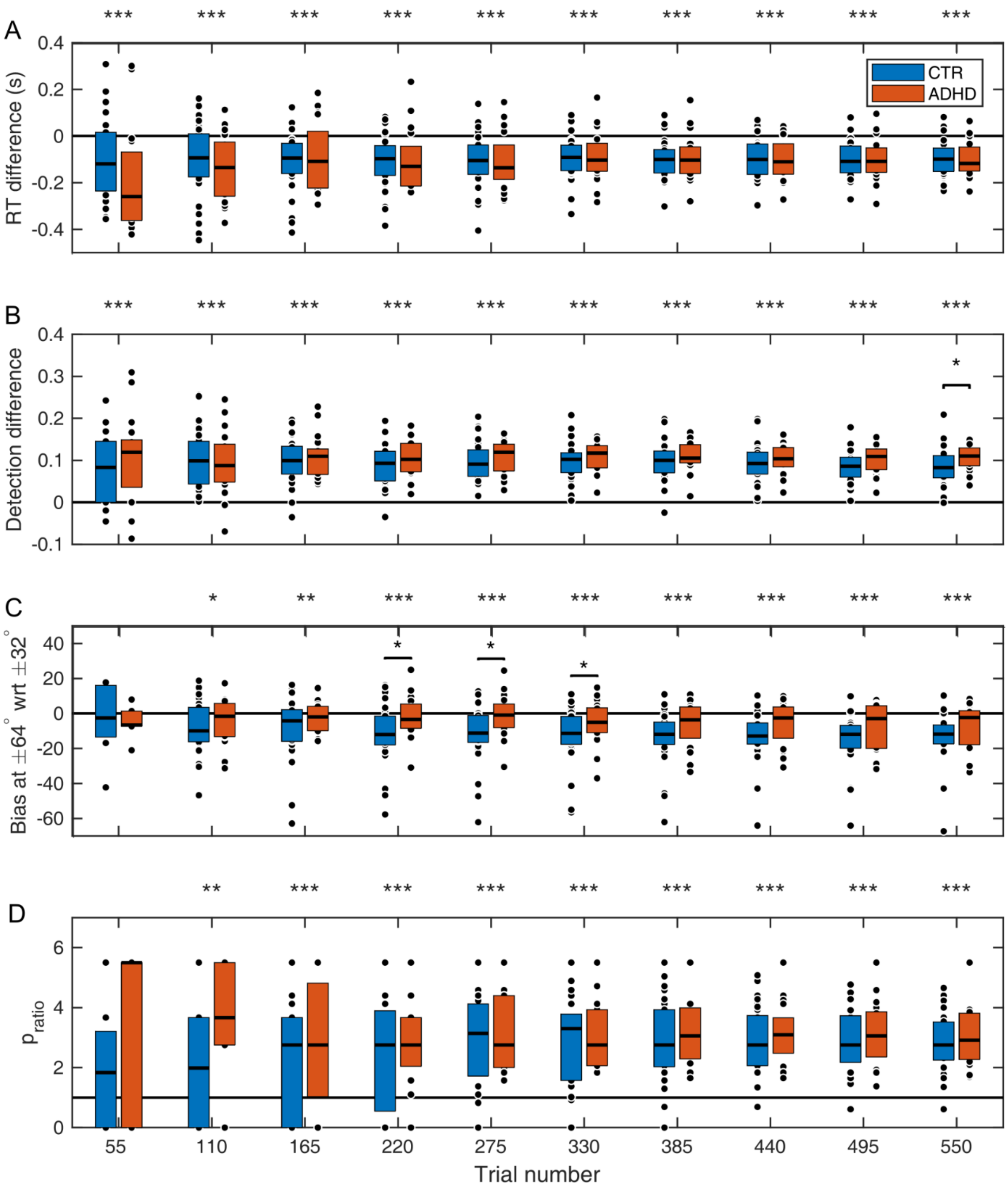

Supplementary Figure 3: Emergence of prior effects in controls (blue boxplots) and ADHD participants (orange boxplots). Cumulative moving averages of median differences between **(A)** estimation RTs at ±32° and RTs at all other directions, **(B)** fraction detected at ±32° and fraction detected at all other directions, **(C)** bias at ±64° with respect to bias at ±32°, and **(D) c**umulative moving averages of the probability ratio of hallucinating predominantly around ±32° on no-stimulus trials. The boxplots indicate 25th and 75th percentiles, the black dash in between indicates the median. The significant effects of prior are indicated above each of the plots (one-tailed Wilcoxon signed rank test), while group differences are indicated within the plots (two-tailed Wilcoxon rank-sum test). *, ** and *** denote significance levels at $p<0.05$, $p<0.01$, $p<0.001$ respectively.

Modelling

Response strategy models ('ADD')

We controlled for the possibility that the task behaviour might be explained by simple behavioural strategies that do not involve Bayesian integration (Laquitaine & Gardner, 2018). This class of models assumed that participants did not combine their expectations with sensory information but relied on either of them alone on any given trial.

The first model, 'ADD1r', assumed that estimations derived from prior expectations were simply sampled from a learnt prior distribution, $p_{prior}(\theta)$, which was parameterized as in Eq (4) - a symmetrical bimodal distribution with nodes at θ_p and $-\theta_p$ and widths of $\sigma_{p.}$ However, on trials when participants perceive motion direction, it was based solely on the sensory input, $p_{likelihood}(\theta_s|\theta_{act}) = V(\theta_{act}, \sigma_s)$.

Putting together the estimations derived from sensory input and the ones derived from learnt expectations, and the possibility of random estimations, the average distribution of estimation responses for a single participant is:

$$p(\theta_{est}|\theta_{act}) = (1 - \alpha)\cdot [(1 - a) \cdot p_{likelihood}(\theta_s|\theta_{act}) + a \cdot p_{prior}(\theta)] * V(0,\sigma_m) + \alpha, \qquad (9)$$

where the asterisk (∗) denotes convolution and 'a' is the probability that on any given trial the sample will be drawn from the prior; following the 'Switching Observer Model' model in Laquitaine & Gardner (2018), 'a' was defined based on the relative precision of the prior: $a = 1/\sigma_p^2 / (1/\sigma_p^2 + 1/\sigma_s^2)$. Just like BAYES and BAYES_P, the resulting 'ADD1r' model had 4 free parameters (θ_p, σ_p, σ_s and α).

The second model, 'ADD2r', was the same as 'ADD1r' except that it had a more complex strategy for trials when participants relied on the prior: instead of sampling from the complete acquired prior distribution ranging from −180° to +180° (Eq. (4)), they sampled only from the negative (−180° to 0°) or the positive (0° to +180°) half, depending on which side of the distribution the actual stimulus occurred on:

$$p_{priorN}(\theta) = V(-\theta_p, \sigma_p) \qquad (10)$$

$$p_{priorP}(\theta) = V(\theta_p, \sigma_p) \qquad (11)$$

Incorporating this into the distribution of estimation responses results in:

$$p(\theta_{est}|\theta_{act}) = (1 - \alpha) \cdot [(1 - a) \cdot p_{likelihood}(\theta_s|\theta_{act})$$
$$+ a \cdot ((1 - b(\theta))\, p_{priorN}(\theta) + b(\theta) \cdot p_{priorP}(\theta)]$$
$$* V(0, \sigma_m) + \alpha, \qquad (12)$$

where asterisk (∗) denotes convolution; b (θ) determines the proportion of trials in which participants sample from either negative or positive parts of the prior distribution, respectively; 'b' could take different values for each of the 5 angles: 0◦, ±16◦, ±32◦, ±48◦, ±64◦). The resulting model had 9 parameters.

Finally, we also considered two variations of the 'ADD1r' and 'ADD2r' models. These were identical to 'ADD1r' and 'ADD2r' except from setting σ_p to zero (i.e. no uncertainty in expectations); that is, on trials when perceptual estimates were derived only from expectations, they were equal to the mode of the learnt distribution. This also meant that 'a' was now estimated as a free parameter. These models are referred to as 'ADD1r_m' and 'ADD2r_m'.

Note that in our previous publications (Chalk et al., 2010; Karvelis et al., 2018, Valton et al., 2019) 'a' was an angle-dependent free parameter which could take different values for each of the 5 angles: 0◦, ±16◦, ±32◦, ±48◦, ±64◦), effectively adding 5 extra parameters to each of the ADD* models. The reduced versions of these models (hence the subscript 'r') presented in this paper were found to outperform the previous versions of the models and for conciseness the old versions were excluded from model comparison.

Parameter estimation

We used the performance in trials with the highest contrast level to estimate motor noise, σ_m, for each individual. We assumed that at this level sensory uncertainty was close to zero ($\sigma_s \approx 0$). To account for lapse estimations, the motor noise was determined by fitting estimation responses at the highest

contrast level to the distribution in Eq. (2) using the actual motion direction, θ_{act}, as the mean. The estimated motor noise for each individual was used in all subsequent model fitting as a fixed parameter.

The free parameters of each model were estimated by fitting the response data from the two staircased contrast levels (~200 trials per participant). For each model with a set of free parameters M, we computed the probability distribution $p(\theta_{est} \mid \theta_{act} ; M)$ of making an estimate θ_{est} given the actual stimulus direction θ_{act}. For the response strategy models, by definition, the $p(\theta_{est} \mid \theta_{act} ; M)$ corresponds to average behaviour in the task (Equations 9 and 12). Bayesian models, on the other hand, explicitly model trial-to-trial variability in the posterior estimate, which in our case is the mean of the posterior (Eq. (6)). To relate this to the behavioural data we built a distribution of 1,000 samples for each presented angle (where each sample is the mean of the posterior obtained via Eq. (6) and perturbed by motor noise via Eq. (7) or (8)).

The parameters were estimated by maximizing the fit of the log likelihood function for the experimental data for each participant individually:

$$M = \text{argmax}_M \left[\sum_i^n \log \left(\mathrm{p}(\theta_{est} = \theta_{i,data} | \theta_i) \right) \right], \quad (13)$$

where $\theta_{i,data}$ is participant's estimation response, θ_i is the actual presented motion direction on the ith trial and n is the number of trials. The maximum likelihood was found using *fminsearchbnd* function in Matlab, by minimizing negative log-likelihood. Parameters α, a and b were bounded between 0 and 1, while θ_p, σ_p and σ_s were bounded from 0 to ∞. To reduce the possibility of convergence at local maxima we performed 20 different initializations with parameter values randomly sampled from the range that we found in our previous work (Chalk et al., 2010; Karvelis et al., 2018; Valton et al., 2019). A set of parameters with the largest log-likelihood was selected as the best fit.

Model Comparison

To compare the model fits we used Bayesian Information Criterion (BIC), which approximates the log of model evidence (e.g., see Burnham and Anderson, 2004):

$$-2 \cdot \log(P(D|M)) \approx BIC = -2 \cdot \log(P(D|M, \Theta)) + k \cdot \log(n) \quad , \tag{14}$$

where M is model, D is observed data and P (D|M, Θ) is the likelihood of generating the experimental data given the most likely set of parameters, Θ; k is the number of model parameters and n is the number of data points (or equivalently, the number of trials). BIC evaluates the model by balancing the goodness of fit with model complexity (i.e. the number of model parameters) to avoid over-fitting. Lower BIC score indicates a better model. We also performed a random effect Bayesian model selection analysis (Rigoux et al., 2014). For this, we used the VBA Matlab toolbox (Daunizeau et al., 2014) and used participant-level BIC as an approximation of the log-model evidence required for the analysis

Parameter recovery

To test the reliability of the parameter estimates of our winning BAYES_P model we performed parameter recovery. Note that this analysis has already been reported in Valton et al. (2019), but we repeat it here for completeness. Parameter recovery allowed us to simultaneously test whether parameters are identifiable (e.g., whether likelihood and prior uncertainty is not correlated and can be distinguished) and whether having ~200 trials (the amount of low contrast trials in our data) for data fitting and using maximum likelihood estimation are sufficient to give reliable results.

First, we generated 100 sets of parameters (i.e. 100 synthetic individuals) by randomly sampling each parameter from a Gaussian distribution that had a mean and variance as the parameter estimates from the collected participant data. Second, for each set of parameters we simulated data for 200 trials with the winning model by randomly sampling from the estimation probability distribution, which, as for the behavioural data, was built from a 1000 posterior means (Eq. (6)), each perturbed by motor noise

(Eq. (8)) Finally, we fitted the winning model to the simulated data. To evaluate the goodness of recovered parameters we computed the coefficient of determination (R^2) for a linear regression, which quantified how well the actual parameters predicted the recovered ones.

We found that the winning BAYES_P model recovered parameters very well, which was reflected in the coefficient of determination (R^2) for all recovered parameters being $R^2 \geq 0.84$ (**Supplementary Fig. 4**).

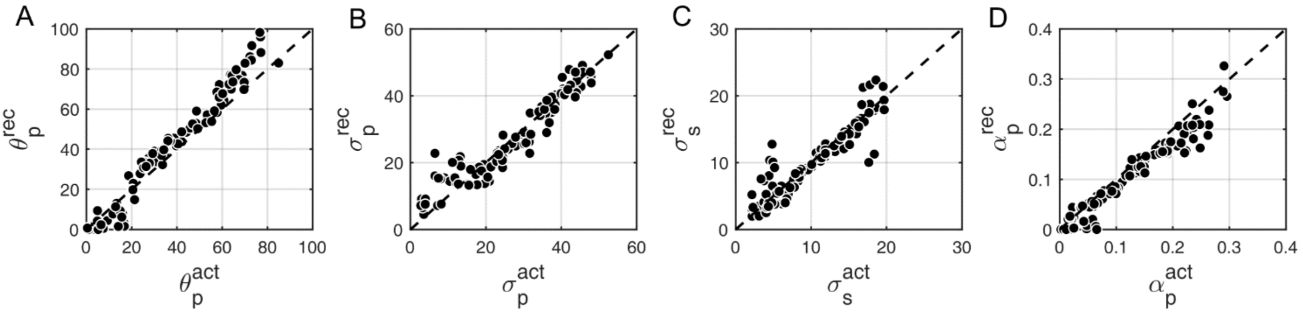

Supplementary Figure 4. Parameter recovery with BAYES_P model. **(A)** θ_p - mean of the prior expectations (R^2 = 0.96), **(B)** σ_p - uncertainty of the prior expectations (R^2 = 0.89), **(C)** σ_s - uncertainty in the sensory likelihood (R^2 = 0.84), **(D)** α_p – prior-based lapse rate (R^2 = 0.94). X-axes – actual parameters used for simulating the data (denoted with the superscript 'act'), Y-axes – recovered parameters (denoted with the superscript 'rec') from fitting the model to the simulated data. The dashed diagonal line is a reference line indicating perfect parameter recovery.

Medication effects

Out of 17 people in our ADHD sample, 10 people were currently taking stimulant medication (6 people were taking methylphenidate, 2 people were taking lisdexamfetamine, 1 person was taking dexamphetamine, and 1 person was taking both lisdexamfetamine and dexamphetamine) and 7 were not taking any stimulants.

We explored the effects of stimulants by looking at the average performance in the task, comparing the subgroup taking stimulants (N = 10) and the subgroup not taking stimulants (N = 7) to controls (N =

30); see figure below. We found no strong evidence for the current use of stimulants having a substantial effect on performance in the task (note that on the day of testing participants did not take their medication). Across all behavioral measures only two results were significant: smaller bias in the medicated group as compared to controls (Z = 2.14, p = .032) and faster reaction times in the unmedicated group as compared to controls (Z = 2.27, p = .023), but these effects did not survive correction for multiple (12) comparisons (**Supplementary Fig. 5**). The interpretation of these effects would be further complicated by uncertainty of their source: are the effects in the group taking stimulants due to consistent use of stimulants, or due to not taking stimulants on the day of testing, or due to possibly more severe ADHD symptoms that underlie the need for stimulants?

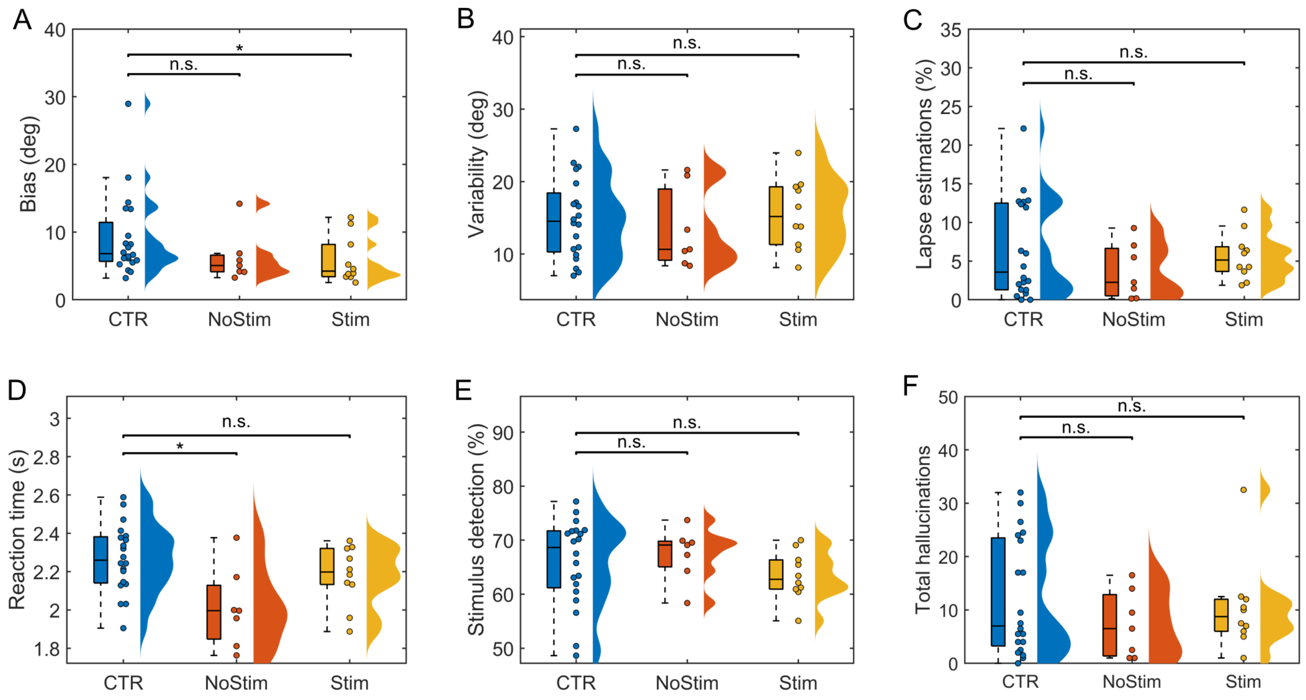

Supplementary Figure 5. Mean performance on **(A-E)** low contrast trials and **(F)** no stimulus trials by controls (blue) ADHD participants that are currently not taking stimulants (orange) and those who are taking stimulants (yellow). **(A)** Mean estimation bias **(B)** estimation variability **(C)** lapse estimations determined using (eq. 1), **(D)** reaction times during the estimation task, **(E)** the fraction of trials in which the stimulus was detected, **(F)** the number of no stimulus trials when stimulus was reported.
