# Supplementary material for: Visual statistical learning and integration of perceptual priors are intact in attention deficit hyperactivity disorder
Source: PLoS One. 2020 Dec 17;15(12):e0243100. doi: 10.1371/journal.pone.0243100 (PMC7746270; doi:10.1371/journal.pone.0243100)
Supplement: Attachment — Submitted filename: reply.docx [file pone.0243100.s003.docx]

**Visual statistical learning and integration of perceptual priors are intact in Attention Deficit Hyperactivity Disorder: Response to reviews**

**Richards et al.**

We would like to thank the reviewers for their comments. In reply, we have made revision to the manuscript. We provide detailed responses to the reviewers below. Reviewers’ comments are in bold italic font, our replies are in normal typeface.

***Reviewer #1:***

***The researchers aimed to test whether there are perturbances in Bayesian inference in individuals with ADHD which might explain the symptoms they experience. During a dot-motion detection task, participants were required to indicate the angle of the movement of the dots, as well as whether the stimulus was present in the first place. Stimuli were mostly like to be moving towards 32 degrees with respect to a reference point, and less likely at 4 other locations either 16 or 32 degrees from the most likely reference point in either direction, creating 5 motion conditions. Behavioural analysis as well as a modelling analysis was used to explore differences between groups. The researchers found a bias towards reporting motion in the most likely direction. However, there were no group differences in these biases. Computational modelling further did not reveal any differences across groups. The paper is well written and the methods are sound. There are a few concerns I would like to raise, but I don’t think they will stand in the way of getting the paper published after some revisions.***

We thank the reviewer for the positive comments.

Major:

***1. The introduction could benefit from a bit more information on how supposedly reduced priors in perceptual inference map onto the symptoms of ADHD specifically, in particular in the presence of a null-finding, and the researchers discussing a similar hypothesis for autism.***

We thank the reviewer for this suggestion. We have now expanded the introduction to make the link between reduced perceptual priors/over-reliance on incoming information and specific ADHD symptoms more explicit.

On page 3:

“First, the failures of behavioral regulation in ADHD could be attributed to disruptions in the formation and/or use of priors, resulting in ascribing excessive precision to incoming information [3]. **Specifically, characteristic symptoms, such as being easily distracted by external stimuli and difficulties maintaining prolonged attention on a task, could be due to excessive precision and therefore attention towards incoming sensory signals.** Indeed, […]”

On page 4:

“ADHD is also associated with a range of sensory modulation issues, including greater difficulties in using **prior** expectations to suppress unwanted saccades and reduced micro-saccade and blink rate around the onset of an anticipated stimulus [15-18]. **Attenuated sensory priors and modulation issues could lead to a barrage of equally pertinent and intrusive sensations that cannot be habituated, resulting in distractibility and impulsive/hyperactive response patterns. Symptoms of inattention and over-activity have been shown to increase linearly with measures of atypical sensation in ADHD and the general population [19-21]**. The evidence […]”

On page 4:

“Second, elevated intra-individual variability has been outlined as a hallmark of ADHD **and is evident in behavioural symptoms such as completing tasks in a muddled way** [43]. **ADHD is associated with notable increases in variability across cognitive domains, including perception** [44-47]. Such findings are suggestive of noisier and less precise distributions at the likelihood and/or prior level in ADHD. **However, it is unclear whether this variability originates from lower-level sensorimotor areas, higher-level cognitive regions, or both.**

***2. The task uses a staircase procedure to control for each subject when the dots are visible. I’m wondering whether this not eliminates a possible effect of sensory precision, and if not why not?***

The staircase procedure ensured that the dots were equally visible for each individual (ensuring the same detection rates). While it is true that stimulus contrast directly affects sensory precision, in our task we distinguish sensory precision about contrast (controlled by the staircases) from sensory precision about the direction of motion. Our findings suggest that neither of these sensory precisions were different in our ADHD sample: both groups converged to the same contrast levels in our task (see Supplementary Figure 2), and there were no differences in sensory precision of motion direction as suggested by the behavioral performance (Fig. 3) and modelling results (Fig. 5).

***3. Given effects of dopamine on the usage of prior expectations in perceptual inference (see Cassidy et al., 2018 for example), I wonder to what extend medication history affects the results. I realise it is a small sample, but it would be good to do an exploratory analysis to see how effects differ between those with a history of stimulant use between those who do not.***

Out of 17 people in our ADHD sample, 10 people were currently taking stimulant medication (6 people were taking methylphenidate, 2 people were taking lisdexamfetamine, 1 person was taking dexamphetamine, and 1 person was taking both lisdexamfetamine and dexamphetamine) and 7 were not taking any stimulants.

We explored the effects of stimulants by looking at the average performance in the task, comparing the subgroup taking stimulants (N = 10) and the subgroup not taking stimulants (N = 7) to controls (N = 30); see figure below. We found no strong evidence for the current use of stimulants having a substantial effect on performance in the task (note that on the day of testing participants did not take their medication). Across all behavioral measures only two results were significant: smaller bias in the medicated group as compared to controls (Z = 2.14, p = .032) and faster reaction times in the unmedicated group as compared to controls (Z = 2.27, p = .023), but these effects did not survive correction for multiple (12) comparisons. The interpretation of these effects would be further complicated by uncertainty of their source: are the effects in the group taking stimulants due to consistent use of stimulants, or due to not taking stimulants on the day of testing, or due to possibly more severe ADHD symptoms that underlie the need for stimulants?

This analysis has now been added to the Supplementary Materials in Medication Effects section. In the main manuscript we refer to it on Page 15:

“Although a washout period was used, it is not feasible to eliminate the cumulative effects of stimulant medication on the brain [70,71]. **Our exploratory analysis of those participants that were currently taking stimulants and those that were not, however, did not suggest stimulant medication to have strong effects on our findings (see Supplementary Material).**”

Minor:

***1. Why is there not a bias found towards the 32 angle in the 0degree condition. It is equally far from the 64 condition where the strongest effect is found, so it is surprising there is no effect in what otherwise is a very similar condition.***

There is no bias at 0 degree angle because the bimodal biases towards –32 and 32 degrees canceled out. Note that while in Fig. 3, which summarizes the main behavioral results, we present data that is ‘folded’ around 0 degree condition, the full range of the presented directions ranged from -64 degrees to 64 degrees (as can be seen in Fig. 5, for instance). Due to the effects being symmetrical with respect to 0 degrees, we folded the data for behavioral data analysis to increase signal-to-noise ratio.

***2. With regards to the task, could you clarify whether participants had to do the estimation and detection task simulataneously or sequantially? Do they require the same button response? How does this work exactly?***

The task was done sequentially. First, participants performed the estimation task by aligning the bar (using a computer mouse to move the bar and a mouse click to confirm their decision). After the estimation task, a new screen appeared with two-alternative forced choice and participants had to move the cursor and click on the left or on the right of the screen to indicate whether they had perceived ‘NO DOTS’ or ‘DOTS'. These details about the task design are depicted and described in Fig. 1.

***3. The results are controlled by AQ scores which makes sense, but is there a correlation in the first place?***

As shown in the figure below, Autism Spectrum Quotient (AQ) and Adult ADHD Self-Report Scale (ASRS) scores were positively correlated across the groups (p=0.012, black); also positively within controls (p=0.041, blue), while within ADHD group it was trending towards negative correlation, but did not reach significance (p=0.097).

We now add those findings to the manuscript on page 8: “Moreover, AQ and ASRS scores were positively correlated across the groups (p=0.012). The measures were positively correlated within controls (p=0.041), while within the ADHD group there was a trend towards a negative correlation that did not reach significance (p=0.097).”

***Reviewer #2: This well-written paper depicts an important study that applies a visual statistical learning task and associated models developed by this research group (Chalk et al, 2010, Parvelis et al, 2018, Valton et al, 2019) to investigate potential impairments in Bayesian inference in ADHD. As the authors emphasize, this study is the first to probe directly Bayesian inference in ADHD and utilize a task and model capable of finding out if ADHD participants have less precise sensory representations or likelihoods or deficits in forming (or using) predictions or priors. This is a valuable endeavor as there is increasing evidence of alterations in Bayesian inference in several neuropsychiatric disorders.***

***This paper has very many additional strengths, both conceptual and methodological: it is one of the few investigations of implicit learning in ADHD, the writing is clear and compelling, the analyses and models are thoroughly presented and executed and thus convincing (i.e, individual data points are presented in several plots, there are 2 different methods for model comparison that lead to the same winning model, parameter recovery is amazing). Overall, I am very happy to see this powerful paradigm applied to ADHD and I believe this article is great and enthusiastically recommend it for publication.***

We thank the reviewer for the positive comments.

***However, beforehand, I would like to see a few aspects developed and unpacked in more detail.***

***Specific comments.***

***1. Overall, I believe the introduction is nicely written and thorough and helps place this work within the broader literature of ADHD and computational psychiatry. However, the authors could also more explicitly allude to the larger literature of reward learning in ADHD and contrast with implicit learning, which can plausibly also be impaired in ADHD, but has been understudied. Something along the lines of: While impairments in reward learning and response have been extensively characterized in ADHD and are thought to stem from dopaminergic dysfunction (for instance, Frank et al, 2007, Silvetti et al, 2013, Kollins and Adcock, 2014, Ziegler et al, 2016, Chevrier et al, 2019), fewer studies have investigated implicit learning in ADHD. The ability to implicitly learn the statistics of a given environment in a short time frame allows for flexible and effective interaction with and function within that environment.***

This is a good point and thank you for providing us with references related reward learning. The section on implicit learning on page 4 has been amended accordingly:

“ADHD is also associated with a range of sensory modulation issues, including greater difficulties in using expectations to suppress unwanted saccades and reduce micro-saccade and blink rate around the onset of an anticipated stimulus [15-18]. **While reward learning deficits have been extensively studied in ADHD and are thought to arise from dopaminergic dysfunction [22-27], implicit learning has received very little attention in ADHD. Implicit learning is thought to play a crucial role in the formation of priors enabling a flexible and efficient interaction with the environment over short timeframes [Wolpert et al., 1995]. Investigations of implicit learning in ADHD are, however, mixed, with some studies finding a difference [29-32], whereas others do not [33-36].** Consistent evidence shows differences in frontostriatal and frontocerebellar circuitry in ADHD **[37-39],** areas implicated in implicit learning **[40-42], lending support to the hypothesis that disruptions in implicit learning and consequently prior formation may account for ADHD symptomatology.**”

***2. page 10. “The evidence of difficulties in implicit learning, crucial for the development of priors, is more mixed in ADHD, with some studies finding a difference [19-22], whereas others do not [23-26]. “ In the discussion, the authors do justice to the possibility that the effects of medication might account for the lack of difference between Controls and ADHD (even if ADHD participants did not take their medication the day of the experiment, the washout period is expected to be longer than that). Are there differences in the medication status of the participants from the studies which found differences in implicit learning in ADHD vs the ones which did not?***

Another good point. On reviewing the studies, there does not seem to be any systematic differences in medication status (i.e. studies included participants regardless of stimulant medication use and there was not a higher or lower percentage of use across the studies). However, limited information (a wash out period was used but no description of the number of participants currently taking stimulant medications) or no information on medication status prevented a full evaluation of this. Where reported, a wash-out period of at least 24 hours was used in the studies that found an effect, whereas for those that did not find an effect, the wash-out period was either 48-hours or psychostimulants were not taken on the day of testing.

***3. page 10. Citations [27-29] seem more suitable to be placed after “ADHD” vs where they are now: “There is also consistent evidence showing differences in frontostriatal and frontocerebellar circuitry in ADHD, areas implicated in implicit learning [27-29]. “ Other citations would support that these areas are also implicated in implicit learning. (i.e. Turke-Browne et al, 2009, Yang and Li, 2012, Leow et al, 2017)***

Thank you for pointing this out and for the additional references related to implicit learning. The sentence on page 4 of the manuscript has been changed accordingly:

“Consistent evidence shows differences in frontostraital and frontocerebellar circuitry in ADHD **[37-39],** areas implicated in implicit learning **[40-42], lending support to the hypothesis that disruptions in implicit learning and consequently prior formation may account for ADHD symptomatology**.”

**4. Results related to Figure 5. The authors are testing several alternative models and the Bayes_P model convincingly captures the data best out of the models tested. The Bayes_P model seems to capture the data well and we can see this visually in Figure 5. I was wondering about two things. First, it seems that in Chalk et al, 2010 and in Karvelis et al, 2018 the Bayes model with a random lapse was able to capture the data very well, but in the Valton et al, 2019 and this paper the Bayes P with the prior-based lapses outperformed the Bayes model, even in Controls. What could account for this difference? One difference I can see is that the lapse parameters were estimated at lower values in Karvelis vs this paper, and thus accounted for a smaller part of the data? Second, while the model fits overlap with the data well, they do not seem quite as impressive as in Chalk or Karvelis. Is this fair to say? If so, what might account for these data sets being harder to fit? Relatedly, this might warrant a bit more caution in the interpretation of the model parameters.**

Thank you for insightful comparisons of results across many of our studies. Firstly, it is important to note that in Chalk et al, (2010) and Karvelis et al., (2018) Bayes_P was not included in the model comparison, as we were not considering this model at the time. We introduced Bayes_P only in Valton et al., (2019). However, refitting data from Chalk et al. and Karvelis et al., also results in Bayes_P being the winning model.

The difference in the lapse rates that can be seen in Karvelis et al. vs this paper is a consequence of using a different model: in Bayes_P model used in this paper the lapses are prior-based and thus can account for a larger number of responses than random lapses in Bayes model used in Karvelis et al. Using the statistical behavioral data analysis, where we must assume random lapse rates, we do not see differences in lapse rate in these two datasets.

It is true that visually the model fits in Chalk et al. and Karvelis et al. appear to be better. In Chalk et al. this is a result of having more data per participant (two sessions of 800 trials), while in this study we had one session of 567 trials per participant. In Karvelis et al., while the number of trials per participant was the same 567 trials, a closer fit is a result of having a larger sample size (N = 83) as compared to the sample size in this study (N = 30 and N = 17). Thus, while a larger sample in Karvelis et al. averages out noise at the group level, the model fits and model parameters in this and Karvelis et al. study are of equal reliability as it depends on the number of trials per participant (the model is fit to each participant separately).

Nonetheless, to alert the reader about the goodness of fit, we have now included the following additions on page 13:

“Both methods suggested BAYES_P model (**Fig. 2**) to be superior (**Fig. 4**). **While parameter recovery analysis showed high recoverability of model parameters (see Supplementary Materials), visual inspection suggested that the BAYES_P fit to the data was not perfect (Fig. 5A-C,E-G), warranting some caution in the interpretation of modelling results.**

**5. Page 19 .Last sentence of results. The authors write “Similarly, ASRS did not correlate with any of these model parameters. “, but do not provide values.**

We have updated the text to provide the values:

“Similarly, ASRS did not correlate with any of these model parameters: **prior mean (𝝉_b = -0.21; p = .076), prior uncertainty (𝝉_b = 0.09; p = .478), sensory uncertainty (𝝉_b = -0.03; p = .827), prior-based lapse rate (𝝉_b = 0.23; p = .056)***”.*

**6. Page 19. Discussion, end of the first paragraph: “there were no group difference in model parameters. “ It is worth spelling out again the model parameters: the mean of the acquired prior, the uncertainty of the prior or the uncertainty of the likelihood. Also, perhaps consider adding that while caution is warranted in the interpretation of individual model parameters, the goodness-of-fits of the Bayes_P model was comparably good across the ADHD and Control groups?**

We have now added the following changes on page 14:

“ The performance of both groups was best described by a Bayesian model of sensory processing (similar to [37, 38, 49). **While the model did not provide an ideal fit warranting some caution in the interpretation of the results, it supported the behavioral data analysis showing no difference between groups in model parameters:** **prior mean, prior uncertainty, sensory likelihood uncertainty and prior-based lapse rate.** ”

**7. Page 21. The authors end with “further testing is warranted in larger, more heterogeneous samples, and with more complex experimental tasks.” In the Chalk paper, the authors write in the discussion: “Future work could investigate this using a more complicated distribution of presented stimuli or statistical learning paradigm that produces slower learning of stimulus expectations (Eckstein et al., 2004; Orba ́n, Fiser, Aslin, & Lengyel, 2008). “ I believe such a discussion point might benefit from a bit more emphasis here, as impairments in ADHD might be revealed in tasks with increased complexity of the prior distribution to be learnt and used, or with slower tasks. The authors’ finding of differences in the influence of the prior over time lends support to this possibility.**

Thank you again for your helpful suggestions. We agree that this is an important point to highlight in the manuscript. We have added the following sentences to page 15:

“Furthermore, differences at the neural level do not always result in observable differences at the behavioral level in ADHD (e.g. [12, 54]). **It is also conceivable that a more complex prior distribution or a task that results in slower acquisition of the prior might allow differences in inferential processes to emerge.** ”
